# Surface engineering to achieve organic ternary memory with a high device yield and improved performance†
†Electronic supplementary information (ESI) available. See DOI: 10.1039/c6sc03986c



**DOI:** 10.1039/c6sc03986c

**Published:** 2016-12-15

**Authors:** Xiang Hou, Xin Xiao, Qian-Hao Zhou, Xue-Feng Cheng, Jing-Hui He, Qing-Feng Xu, Hua Li, Na-Jun Li, Dong-Yun Chen, Jian-Mei Lu

**Affiliations:** a College of Chemistry, Chemical Engineering and Materials Science , Collaborative Innovation Center of Suzhou Nano Science and Technology , National United Engineering Laboratory of Functionalized Environmental Adsorption Materials , Soochow University , Suzhou 215123 , PR China . Email: jinghhe@suda.edu.cn ; Email: lujm@suda.edu.cn

## Abstract

Squaraine molecules deposited on indium tin oxide (ITO) substrates modified with phosphonic acids crystalize more orderly than do those on untreated ITO. The as-fabricated electro-resistive memories show the highest ternary device yield observed to date (82%), a narrower switching voltage distribution, and better retention as well as resistance uniformity.

## Abstract 2

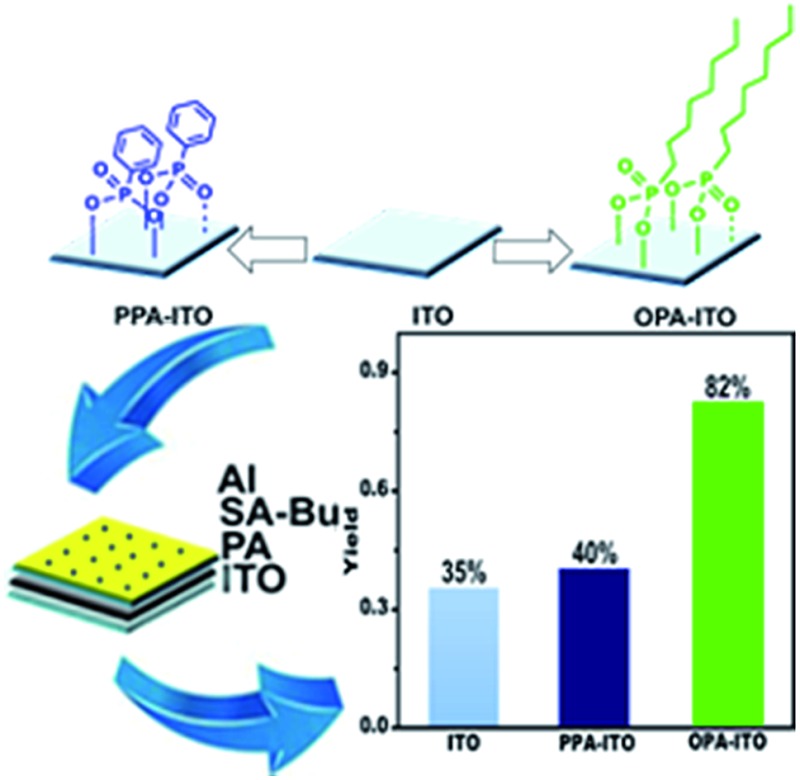
Organic memories fabricated on surface-engineered indium tin oxide show the highest ternary yield (82%) to date and better performance.

The information explosion in modern society requires increasingly higher densities in data storage, which is gradually becoming difficult to achieve through traditional binary memory techniques. Resistance switching random access memory (RRAM),^1^ as a promising high-density memory solution, is gradually attracting both fundamental and industrial interest.^2–4^ RRAMs consist of two electrodes sandwiching a thin layer of active material that, under an external electric field, possesses different conductive states, which are considered “0” or “1” for information storage.^4^ In addition, due to their simple three-layers structure, RRAMs can be used for three-dimensional stacking and can dramatically expand the information density.^5^ Most importantly, if multiple conductive states exist in the active materials, each memory cell can store more than two states, such as “0”, “1”, “2”…. This multilevel RRAM technique is expected to result in a revolutionary enhancement in the information density from 2^*n*^ to *X* ^*n*^ (where *n* is the cell density and *X* is the state number of each cell).^6^

After decades of intensive research, many inorganic oxides,^7^ polymers^8^ and small molecules^9,10^ have been found to exhibit multilevel resistive switching behaviours and are thus considered candidate active materials for multilevel RRAMs. However, to enable industrial use, RRAM devices must show good reproducibility, including a high multilevel memory device yield and narrow distribution of OFF/ON1/ON2 switching voltages. Unfortunately, numerous studies have explored new RRAM active materials based on the measurements on individual devices, but statistical data on a large batch are rarely reported.^4^ Our recent statistical work^9,11–13^ demonstrated that the ternary memory yield of organic RRAMs is generally low (∼50%), despite the effectiveness of the molecular design in improving the ternary yield and switching voltage distributions. The frustration of material innovation has urged us to seek other strategies for improving device reproducibility.

In the fields of organic light emitting diodes (OLEDs) and organic photovoltaics (OPVs), it is widely accepted that the surface energy matching and work function compatibility at the interfaces between the electrodes and the organic layer are critical to the device performance.^14,15^ Therefore, surface engineering of the substrate, particularly of indium tin oxide (ITO) glass, is widely employed.^16^ Among the various ITO surface modification methods, self-assembly of the phosphonic acids (PAs) monolayer was demonstrated to be the most effective approach due to the abundance of possible choices for the terminal functional groups,^17,18^ stronger binding strength to ITO than that of siloxanes and carboxylic acids, high stability under ambient conditions, and simple modification and processing.^17–21^ Surface modification by PAs usually gives rise to high performance OLEDs and OPVs; however, it has not been introduced into organic memories.

In this paper, we demonstrate for the first time that surface engineering is an effective strategy beyond molecular design for achieving high ternary RRAM device yields. When the bottom ITO substrate was modified by PAs to form a self-assembled monolayer, deposited organic molecules grown on the modified substrate crystalize in a more orderly fashion than on the untreated ITO. The device yield of the ternary RRAMs increases to 82%, which is the highest value obtained to date, to the best of our knowledge. Our work will inspire the application of surface engineering to the fabrication of organic memories with high reproducibility and will promote the use of organic memory in practical applications.

A new squaraine molecule, 2-((4-butylphenyl)amino)-4-((4-butylphenyl)iminio)-3-oxocyclobut-1-en-1-olate (SA-Bu), was synthesized and used as the active material in our RRAM devices. The molecular structure of SA-Bu is shown in Fig. 1a, and the detailed synthesis and characterization of SA-Bu are described in the Experimental section, Fig. S1 and S2.† Squaraines show resonance-stabilized zwitterionic characteristics originating from their squaric acid rings and amine moieties. Therefore, they exhibit strong absorption and emission in the near-IR range, low band gaps and high photostability, which can lead to wide applications in optical recording,^22^ solar cells,^23^ electrophotography, chrom/fluorogenic sensing,^24^ thin film transistors^25^ and gas sensors.^26^ These properties guarantee that SA-Bu has unique electronic properties and is thus likely to be appropriate for RRAM device fabrication.

**Fig. 1:**
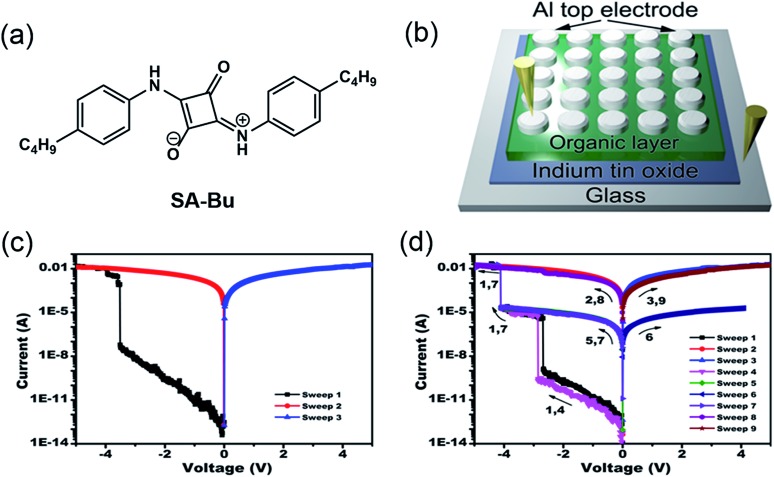
(a) Molecular structure of SA-Bu, (b) diagram of the prototype sandwich device, and typical current–voltage (*I*–*V*) characteristics of the (c) binary and (d) ternary devices.

Our memory devices were fabricated through the thermal evaporation of the SA-Bu layer under vacuum onto ITO substrates followed by the deposition of aluminium top electrodes, as shown in Fig. 1b. The film thickness of the organic active layer and aluminium electrode are approximately 80 and 100 nm, respectively (Fig. S3†). Using a general degreasing procedure, prior to device fabrication, the ITO substrates (denoted as the original ITO hereafter) were cleaned by sonication in various organic solvents and pure water sequentially. The devices fabricated on these ITO substrates show both binary and ternary memory behaviours, as demonstrated by the *I*–*V* characteristics shown in Fig. 1c and d, respectively. In Fig. 1c, when the voltage applied to the bottom and top electrodes was swept from 0 V to –5.0 V, an abrupt increase in the current from 6.2 × 10^–8^ A to 3.8 × 10^–3^ A was observed at the switching threshold voltage of –3.8 V, indicating the transition from a low conductive (OFF) state to a high conductive (ON) state. The ON state is retainable under the following sweeps from 0 to 5 and 0 to –5 V. In Fig. 1d, as the voltage increases, two abrupt jumps were observed at –2.8 and –4.0 V for a ternary device. The current ratio of these three states is 1 : 5.2 × 10^3^ : 1.5 × 10^7^, indicating the transition from a low conductivity (OFF) state to an intermediate conductive (ON1) state and further to a highly conductive (ON2) state. This OFF/ON1/ON2 transition can be considered a “writing” process. The ON2 state was also sustainable during the negative sweeps from 0 to –5.0 V (sweep 2) and positive sweeps from 0 to 5.0 V (sweep 3). When applying a lower negative voltage from 0 to –4.0 V to another cell of the device, the OFF-to-ON1 transition occurred at approximately –2.9 V (sweep 4). The subsequent forward and backward scans from 0 to –4.0 V (sweeps 5 and 6) indicate that the device remained in the ON1 state. Furthermore, the ON1-to-ON2 transition was obtained in the same cell at –4.1 V by sweep 7. Finally, this ON2 state could be maintained during the following negative and positive sweeps (sweeps 8 and 9). In short, the SA-Bu devices deposited on the original ITO substrates show both binary and ternary WORM (Write-Once-Read-Many times) memory behaviours. It should be noted that for the industrial use of multilevel RRAM, the ratio of ternary to binary in a device batch should be as high as possible to avoid reading/writing errors. Furthermore, the stabilities of the SA-Bu-based memory devices on ITO, PPA–ITO and OPA–ITO substrates were studied using the continuous voltage stress and readout test at room temperature and 100 °C, respectively (Fig. S4†). During these tests, no obvious degradation was observed in any of the three states for at least 10 000 s, despite the small resistance fluctuation.

To improve the properties of the ternary memory devices, we modified the ITO substrate by PAs self-assembly, as shown in Fig. 2a. Here, the tethering by the aggregation growth procedure (T-BAG)^27^ was applied to modify the ITO substrate to obtain the highest surface coverage of the phosphonic acids.^28^ Two types of ITO substrates were prepared, denoted PPA–ITO and OPA–ITO, corresponding to the ITO modified by phenylphosphonic and octylphosphonic acids, respectively.

**Fig. 2:**
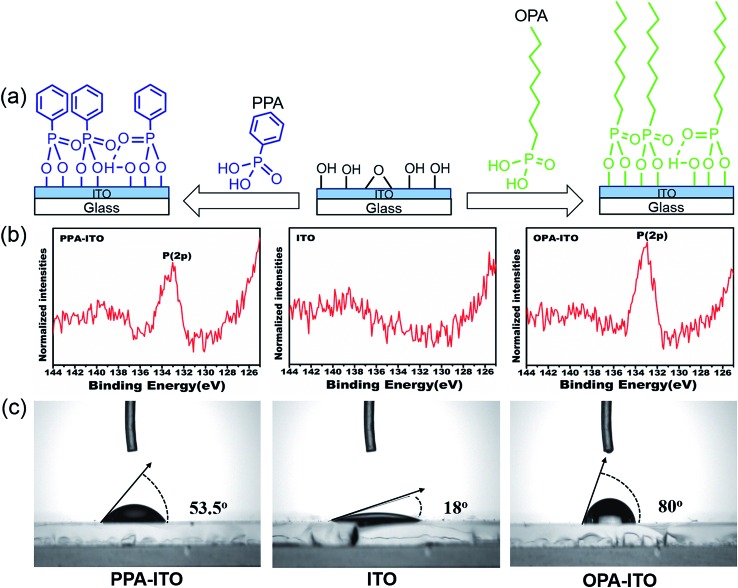
(a) Process of surface modification by phosphonic acids on the surface of ITO glass; (b) XPS spectra of P(2p) of PA-modified ITO substrates; (c) wettability tested in air.

The XPS data for PPA–ITO, OPA–ITO and original ITO are displayed in Fig. 2b. For PPA–ITO and OPA–ITO, the P 2p peak could be found at the same position at 133.4 eV, but no distinct feature is observed in this range for the film on the original ITO. The peak position is in good agreement with the previous studies, confirming that the PAs are successfully self-assembled onto the ITO substrates.^20,28,29^ In addition, the change of the wettability of these surfaces supports the successful modification of the ITO surfaces. The original ITO surface consists of hydroxyl and carboxyl groups^20,29^ and thus shows good wettability toward water, with a contact angle of 18°. After the PAs treatment, the contact angles of PPA–ITO and OPA–ITO increase to 53.5° and 80°, respectively (Fig. 2c).^28^ The PPA–ITO and OPA–ITO surfaces exhibit typical hydrophobic properties because the weak polar phenyl and octyl groups in PAs are pointing outward (Fig. 2a). In short, the XPS and wettability results are in good agreement with those of previous work, confirming that octylphosphonic and phenylphosphonic acids were successfully adsorbed on the ITO surface.

The sandwich-structured organic memory devices (OMDs) were fabricated on the PAs-treated ITO substrates under the same conditions as on the original ITO. As shown in Fig. 3a, fifty units of each type of device were tested to survey the reproducibility and variation trends upon surface engineering. All OMDs still exhibited binary/ternary WORM memory behaviours, as observed in Fig. 1c and d. However, the ternary device yield, as defined by the proportion of the ternary memory to the total devices, was significantly improved. The ternary device yield on the original ITO is only approximately 35%. In contrast, after surface engineering, this yield rises remarkably to 40% (on PPA–ITO) and 82% (on OPA–ITO). To the best of our knowledge, 82% is the highest value that has been reported based on the statistics of a large sample size. Because this is the first report of using PAs modification to improve memory performance, a higher ternary device value may be achieved in the near future if other, better organic molecules are employed.

**Fig. 3:**
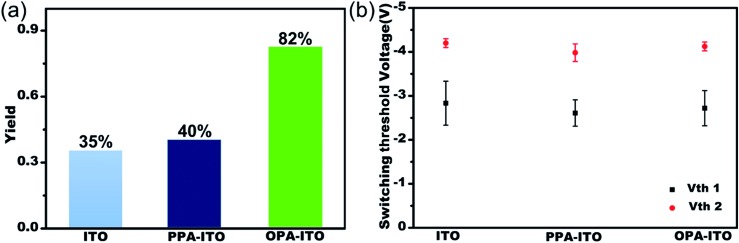
(a) Ternary yield based on three types of substrates; (b) statistics for switching threshold voltages of both the *V*_th_1 state and *V*_th_2 state of ternary devices with error bars (variance).

The switching threshold voltages (*V*_th_s) were also slightly reduced by using this surface engineering technique. As shown in Fig. 3b, the threshold voltages, *V*_th_1 and *V*_th_2, of all three types of ITO substrates are well separated with small deviations. After the modification, both *V*_th_1 and *V*_th_2 of the devices are slightly reduced, from *V*_th_1/*V*_th_2 = –2.83/–4.20 on ITO and from –2.61/–3.98 on PPA–ITO to –2.72/–4.12 V on OPA–ITO (see the detailed distribution histograms in Fig. S5†). Compared to the devices on ITO and PPA–ITO, the *V*_th_1 of OMDs on OPA–ITO shows the narrowest distribution as indicated by the error bars; this is also critical for avoiding reading/writing errors in the RRAM arrays.

In addition, after surface modification, the resistance stability during the retention test is improved. The data presented in Fig. S4† show that the resistance of each state of the device based on the OPA–ITO substrate is more stable at both room temperature and 100 °C (Fig. S4†).

Because each RRAM state is read by measuring the resistance (or voltage/current from the *I*–*V* curves), we further investigated the effect of the surface modification on the resistance uniformity over a batch of devices. The ratio of the highest (lg *R* > 10) and lowest (lg *R* < 2.5) resistance greatly increases after the surface modification such that the OFF and ON2 states could be more facilely distinguished. Further, the middle resistance state (5.25 < lg *R* < 8.75) was distributed more narrowly and thus showed a better separation from the OFF and ON1 states, lowering the writing and reading errors (Fig. S6 and S7† and related description).

The good memory performance described above originates primarily from the unique electronic properties of the SA-Bu molecule. According to the cyclic voltammetry (CV) spectra shown in Fig. S8a, † the HOMO/LUMO values were determined to be –5.35/–2.59 eV. The maximum UV-visible absorption peaks in the DMSO solution and film are located at 402.5 nm and 349.5 nm, respectively, as shown in Fig. S8b.† This peak could be assigned to a π–π* transition within the molecular backbone.^30^ Compared to the spectrum in the solution, the major peaks in the film state show a large hypochromic shift by 53 nm. This shift in the film state indicates severe H-aggregation,^2,31^ which favours charge carrier transport between the neighbouring molecules in the film.^32^ In addition, the onset of the UV-absorbance is located at 445.1 nm, corresponding to the small optical band gap of 2.76 eV.

The intermolecular interaction and crystallinity of the organic layer are other key factors that enable the achievement of a high ternary device yield, as inferred from our previous studies.^11,12^ We collected the X-ray diffraction (XRD) patterns for the SA-Bu powder prior to the deposition and for the films deposited on the three types of ITO substrates (Fig. S9†). Multiple strong diffraction peaks can be observed in the XRD patterns of the SA-Bu powders and all films, suggesting that the SA-Bu molecules exhibit strong intermolecular interactions and are highly crystallized in the solid state. This is due to the occurrence of the strong intramolecular charge transfer in the squaraines exhibiting resonance-stabilized zwitterionic properties. The uneven charge distribution in SA-Bu causes the neighbouring molecules to interact more effectively.^33^

In contrast, the SA-Bu stacking manner is affected by the substrate and shows large differences between the film state and the powder state. In the small angle range (2*θ* ∼ 0–10°), the SA-Bu powder prior to the evaporation shows two strong features at 2*θ* ∼ 3.9° (*d* = 22.5 Å), 4.7° (*d* = 18.9 Å) (Fig. S9†). In these films, the two peaks that are originally present in the powder state disappear, but all of the SA-Bu films show only one sharp peak at 2*θ* ≈ 4.3°. Considering the molecular length of SA-Bu (∼23 Å), this enhanced feature should be attributed to lamellar stacking parallel to the substrate with an interlayer distance of approximately 20.8 Å, induced by the substrate.

To further reveal the stacking manner of SA-Bu in the films, grazing-incidence small-angle X-ray scattering (GISAXS) analysis was adopted to determine the mosaicity (the measure of spreading of crystal plane orientations) of the films. In the GISAXS measurements (Fig. 4 and S10†), all films were impinged by a monochromatic X-ray beam with a very small incident angle, and the crystallite information could be obtained from the shape and intensity distribution of the peaks in the two-dimensional reciprocal (*q*_*xy*_ – *q*_*z*_) space.^34,35^ In Fig. 4a, GISAXS patterns of all films on the ITO substrates show two arcs along the *q*_*z*_ direction, indicating a highly ordered lamellar structure parallel to the ITO substrate, illustrated in Fig. 4d. The arcs imply interlayer spacing distances of 24.9 Å (*q*_*z*_ = 0.25 Å^–1^) and 22.4 Å (*q*_*z*_ = 0.28 Å^–1^) on the original ITO substrate, 25.9 Å (*q*_*z*_ = 0.242 Å^–1^)/22.4 Å (*q*_*z*_ = 0.28 Å^–1^) on PPA–ITO and 25.7 Å (*q*_*z*_ = 0.240 Å^–1^)/22.9 Å (*q*_*z*_ = 0.274 Å^–1^) on OPA–ITO.^35–37^ The film on the PPA–ITO substrate also exhibits two arcs but with a narrower angular distribution, indicating that the mosaicity of the crystallites is improved. Notably, in the case of OPA–ITO, two arcs gradually merge into a single arc, corresponding to highly aligned domains (Fig. 4f).^35,36^ It is plausible that these two arcs reflect two stacking structures, the preferential growth of which is induced by the OPA-modified surface.

**Fig. 4:**
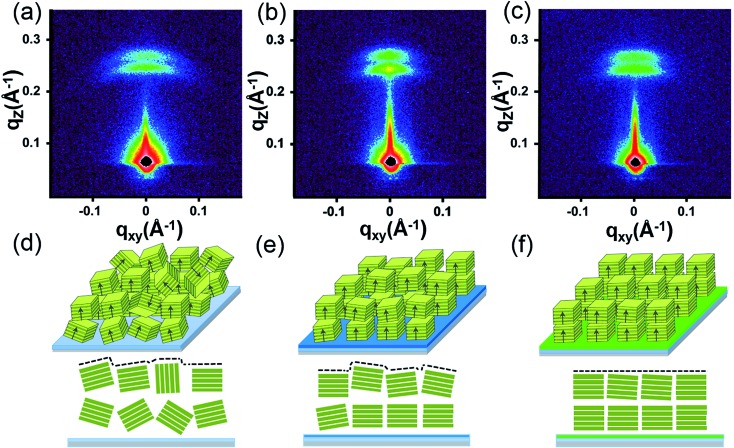
(a)–(c) GISAXS pattern of SA-Bu evaporated on ITO, PPA–ITO and OPA–ITO substrates and (d)–(f) the proposed crystallite stacking models, respectively.

On the macro-scale, the device performance in organic electronics is highly correlated with the surface morphology of the active layer. Fig. 5a shows atomic force microscopy (AFM) images of the film grown on the untreated ITO substrate. The surface root-mean-square (RMS) roughness of the SA-Bu film is 0.869 nm, with a relatively strong phase separation. The surface morphology becomes smoother with a roughness of 0.715 nm (Fig. 5b) on PPA–ITO. In particular, a well-defined isotropic “granular” structure with a RMS of 0.268 nm is formed on OPA–ITO.

**Fig. 5:**
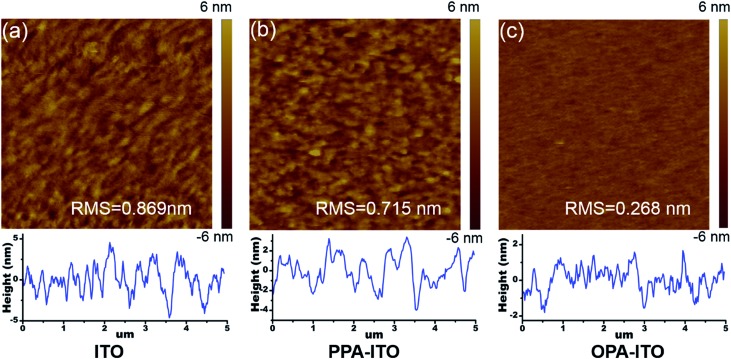
Morphology characterization of non-contact mode AFM topographic images and corresponding cross-section profiles of SA-Bu films evaporated on ITO (a), PPA–ITO (b) and OPA–ITO (c) substrates, respectively. All image sizes are 5 μm × 5 μm.

Based on the above CV, UV, XRD and GISAXS evidence, the variation trends of the crystallite mosaicity and surface morphologies could be understood from the interaction between the outermost surface groups from the substrate and the weak polar butyl groups from SA-Bu. SA-Bu molecules have a rigid and conjugated core that is terminated by two butyl chains. This structure prefers to form a layer-by-layer stacking structure, where cores stacking *via* π–π stacking and tailoring alkyl chains attract each other.^38^ On the original ITO substrate, highly polar hydroxyl and carboxyl groups are present that repel the insertion of the first layer of the weak polar butyl chains, thus disturbing the ordered assembly of the SA-Bu molecules.^15^ The PPA-modified ITO substrate has phenyl rings with moderate polarity on the top and thus shows moderate affinity towards the insertion of the SA-Bu butyl chains.^17,28^ Finally, the OPA-modified ITO surface has self-assembled octyl chains uniformly pointing outward, favouring the insertion of ordered layers of butyl chains.^17,19^ In other words, the octyl chains induce the ordered aggregation of the first few layers of SA-Bu through the alkyl–alkyl chain interaction. The uniformly oriented SA-Bu molecules also tend to form larger crystallites with reduced mosaicity and smoother morphologies.

It is widely accepted that reduced mosaicity and smoother morphologies of organic films improve the quality of organic films in organic field-effect transistors ^39^and solar cells.^26,40^ In our case, the improvements of the ternary device yield and threshold voltages also originate from the reduced mosaicity and smoother morphologies using our extended charge trap mechanism.^12^ In our previous study, the charge trapping mechanism was extended by invoking the polarization field of charged traps to explain the organic multilevel electrical switching. Briefly, small organic molecules often crystallize imperfectly through weak intermolecular interactions, leading to the existence of a high density of localized states (charge traps). These traps that immobilize the charges during charge transport will polarize the surrounding molecules, which will, in turn, become more conductive and accelerate the charge accumulation and further polarize more molecules. Such a positive feedback effect will gradually accumulate charge traps and create conductive tunnels that connect the electrodes, thereby producing current jumps under certain values of the external electric field. If two trap levels exist, they will be filled sequentially once a sufficient electric field is applied. Therefore, there will be two conductive jumps, corresponding to the OFF/ON1 and ON1/ON2 switching, producing ternary memory behaviours. After removing the external electric field, if the charges at these traps cannot be released, the conductive channels will be sustained and will be detected by the next scan, leading to the WORM behaviours.

In our polycrystalline film, the conductive tunnels must pass through the grain boundaries connecting the grains in series, and charge transport must overcome an extra boundary barrier by consuming part of the voltage (electric field). Given the constant external voltage, if too much voltage is consumed, the residue electric filed applied to each grain is too small to fill the deep traps. In this work, after the substrate modification by PPA and OPA, the molecular mosaicity in the film is greatly reduced, and the crystallites with parallel orientations align more uniformly, thus improving the contacts at the boundaries between neighbouring crystallites. Therefore, in most surface-modified devices, the voltage distributed on the grain is sufficient to fill both trap levels and thus generate ternary memory behaviours. In addition, the smoother surface morphology also generally improves the contact between the top electrode and the active layer, thereby reducing the contact resistance.^41^ Both of these changes help to improve the ternary device yield and lower the switching voltages.

## Conclusions

In summary, we have demonstrated surface engineering of the ITO substrate as an efficient method for adjusting the molecular stacking in a SA-Bu thin film. Following the modification of the ITO substrates by phosphonic acid, the intermolecular stacking interactions as well as the mosaicity of crystallites are greatly improved, thus improving both the contact between the grain boundaries and the performance characteristics of organic ternary RRAMs, including the high ternary device yield, narrow distribution of switching voltage, better retention time and better resistance uniformity. Although these OMD parameters still need to be further optimized for industrial use, our results update the understanding of how intermolecular stacking affects the performance of OMDs upon substrate modification and offer an effective strategy to achieve high-performing organic memory devices.
