# Supplementary material for: Surface engineering to achieve organic ternary memory with a high device yield and improved performance
Source: Chem Sci. 2016 Dec 15;8(3):2344–51. doi: 10.1039/c6sc03986c (PMC5364995; doi:10.1039/c6sc03986c)
Supplement: Supplementary file 1 [file SC-008-C6SC03986C-s001.pdf]

Electronic Supplementary Material (ESI) for Chemical Science.
This journal is © The Royal Society of Chemistry 2016

Supporting Information

Surface Engineering to Achieve Organic Ternary Memory with A High Device Yield and Improved Performance

Xiang Hou, [a] *Xin Xiao,* [a] *Qian-Hao Zhou,* [a] *Xue-Feng Cheng,* [a] *Jing-Hui He,* *[a] *Qing-Feng Xu,* [a] *Hua Li,* [a] *Na-Jun Li,* [a] *Dong-Yun Chen* [a] *and Jian-Mei Lu**[a]

Xiang Hou, Xin Xiao, Qian-Hao Zhou, Xue-Feng Cheng, Prof. Jing-Hui He, Prof. Qing-Feng Xu, Prof. Hua Li, Prof. Na-Jun Li, Prof. Dong-Yun Chen and Prof. Jian-Mei Lu
College of Chemistry, Chemical Engineering and Materials Science, Collaborative Innovation Center of Suzhou Nano Science and Technology, Soochow University, Suzhou 215123, PR China; National United Engineering Laboratory of Functionalized Environmental Adsorption Materials, Soochow University, Suzhou 215123, PR China

E-mail: jinghhe@suda.edu.cn;lujm@suda.edu.cn

Experimental Section

The synthesis and characterization of SA-Bu.

Squaric acid (1.00 g, 8.76 mmol) and 4-butylaniline (2.61 g, 17.52 mmol) in n-butanol/toluene(40 mL, 1/1 in vol)[1]. The mixture was refluxed for 12 h. The afforded precipitate was collected by filtration and washed by $CHCl_3$ and hexane to offer SA-Bu as orange solid in 99% yield.**^{1}H NMR** (400 MHz, DMSO-d_6): δ 9.95 (s, 1H), 7.38 (d, *J*=4.0Hz, 4H), 7.15 (d, *J* = 4.0Hz, 4H), 2.51 (t, *J* = 4.0Hz, 4H), 1.51 (m, *J* = 4.0 Hz, 4H), 1.28 (m, *J* = 4.0 Hz, 4H), 0.87 (t, *J* = 4.8 Hz, 6H).

Modification of ITO surface with Phosphonic Acids.

The ITO slides were immersed horizontally on top of a clean sample holder in a 1 mmol/L of phosphonic acids in ethanol. About 70% (in vol) of the modifying solution was underneath the sample. The solvent was allowed to evaporate until the volume fell below the substrate. The ITO substrates were then sonicated in absolute ethanol to remove the physisorbed phosphonic acids. After sonication, the ITO samples are annealed in a vacuum oven at 65 °C for 6 h. The dry ITO glasses were sonicated again for 30 minutes in a trimethylamine/ethanol solution (1/20 in volume ratio), followed by washing in absolute ethanol.

Fabrication of the memory device.

SA-Bu was thermally evaporated onto the surface of the modified ITO under a pressure of 10^{-4} Torr. A layer of Al was thermally evaporated and deposited onto the organic surface at a pressure of about 3×10^{-6} Torr through a shadow mask to yield top electrodes with a thickness of around 100 nm and area of 0.0314 mm^2.

Instrumentation and Characterizations.

NMR spectra were measured on INOVA 400 MHz spectrometers. UV-vis absorption spectra were measured at room temperature with a Shimadzu UV-3600 spectrophotometer. Cyclic voltammograms (CVs) were collected by a CorrTest CS Electrochemical Workstation analyzer in a solution of 0.1mol/L tetrabutylammonium hexafluorophosphate ($TBAPF_6$, used as electrolyte) in DMSO at a sweeping rate of 100 mV/s, with the assistances of an ITO working electrode, a Ag/AgCl reference electrode, and a Pt wire counter electrode. Atomic force microscopy (AFM) measurements were performed using an MF P-3DTM (Digital Instruments/Asylum Research) AFM instrument. XRD measurements were carried out with a Multiple Crystals X-ray Diffractometer (X'Pert PRO, PANalytical). The fabricated devices were characterized under ambient conditions using 4200-SCS semiconductor characteristic analysis system from Keithley.

Resistance uniformity analysis

From a view of real application, resistance uniformity requires using a certain voltage (or in a writing window) to write a batch of devices to change their resistances, that should could be classified by a well-defined state such as “OFF”, “ON1” or “ON2” etc. This means for writing in any writing voltage window such as for “OFF”, “ON1” or “ON2”, the written resistance range (windows) should also be as narrow as possible. Otherwise, once the resistance ranges of each state overlaps, they will be mistakenly readout, leading to storage error.

We deduced all resistances at each voltage (R=I/V) from all I-V curves. The logarithm of resistance “lgR” was plotted using a two-dimensional contour map to show the resistance distribution of all devices in **Figure S6**. It can be seen from **Figure S6** that all devices have large variation of resistance, reflecting the switching behaviors. To further survey the impact of our surface modification methods on the resistance distribution, we sorted the resistance at each particular voltage and replotted the figure in the similar manner as shown in **Figure S7**. Conclusion regarding to the voltage window and resistance could be drawn by investigating the color zones as following.

First, after surface modification, the highest resistance ($lgR>10$, Red zones) and lowest zone (Deep blue zone, $lgR<2.5$) all grows. This means the resistance distribution become more diverse, which is beneficial to effectively separating the writing voltage windows of OFF and the lowest conductivity state (ON for binary or ON2 for ternary).

Second, for example, judging from the color distribution of blank-ITO devices, two green ribbons (middle resistance range, $5.250<lgR<8.750$) spread overall the whole voltage range, indicating that these middle resistances could be written and later read out by any voltage. So it is not suitable for blank-ITO as a ternary device. In contrast, the green ribbons after surface modification by OPA or PPA become much steeper. Thus, it is possible to setup a middle state window (ON1) to write resistances which are well separated by other two writing windows (OFF and ON2 in Figures S7b and S7c).

Finally, we noted that the resistances of each window are not perfectly separated from its neighboring states, which has been a general problem in the field of organic multilevel devices as we mentioned in the introduction. We will do more work to solve it in our future study.

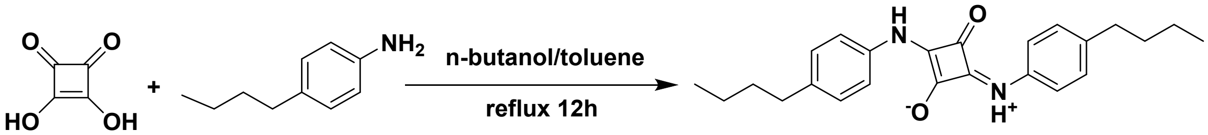

Figure S1. Synthetic routes of SA-Bu.

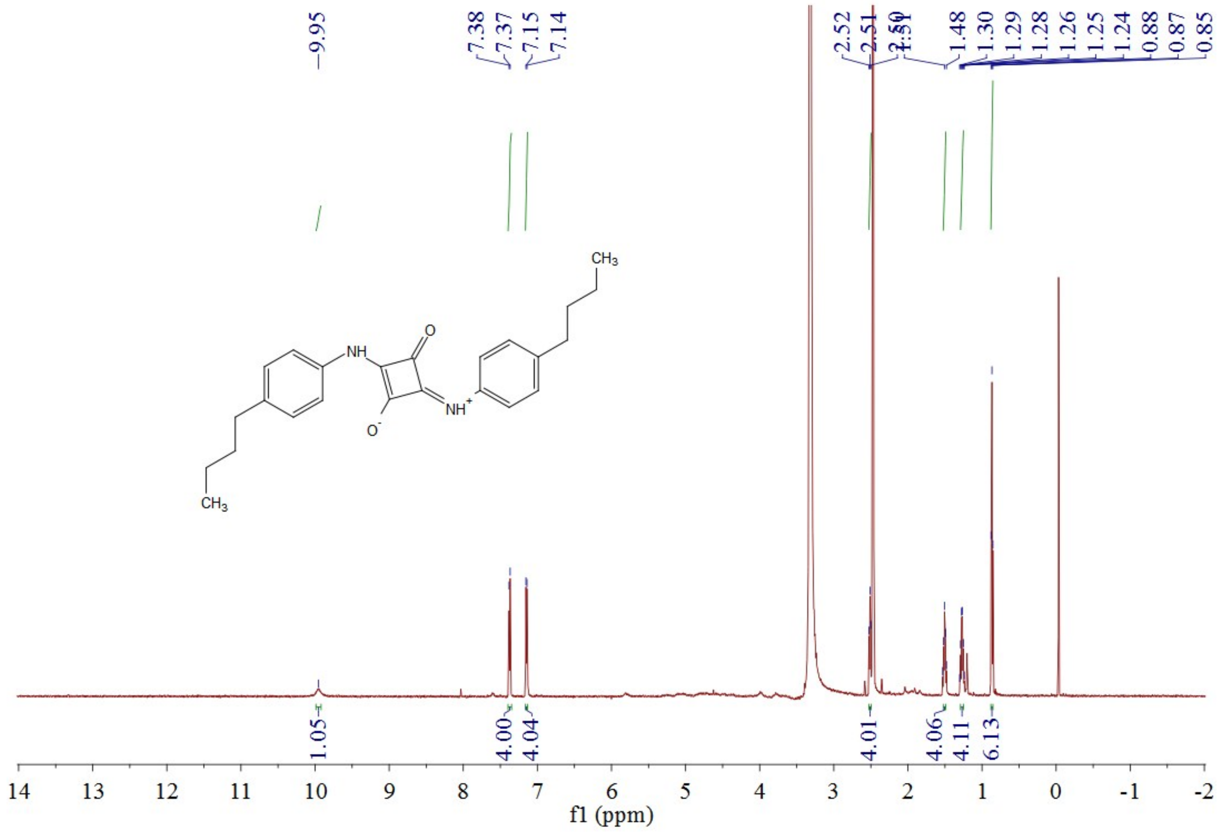

Figure S2. ^{1}H NMR spectrum of SA-Bu in DMSO.

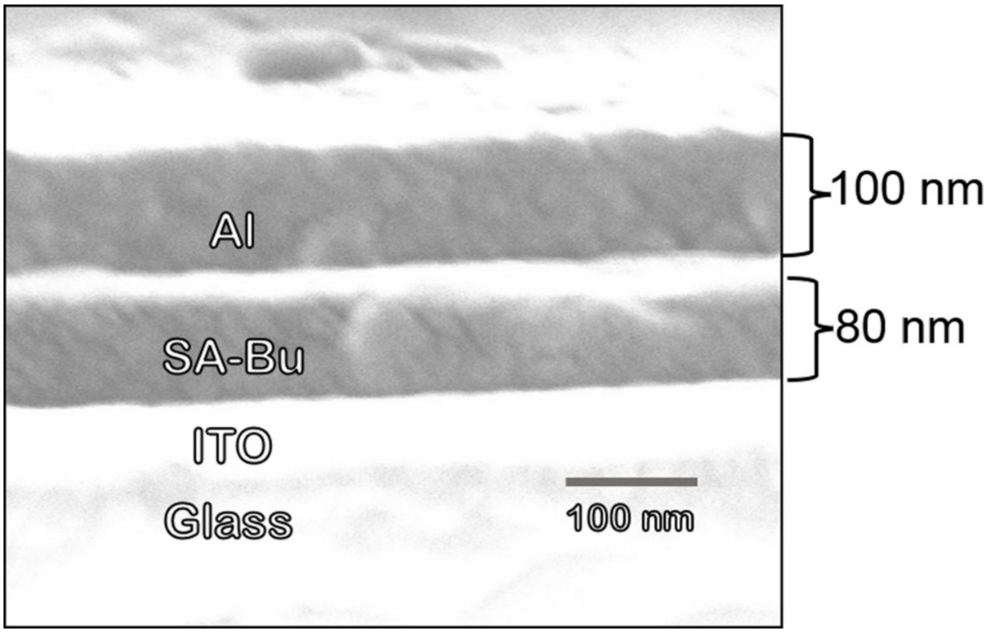

Figure S3. SEM images of a cross-section view of the fabricated RRAM device.

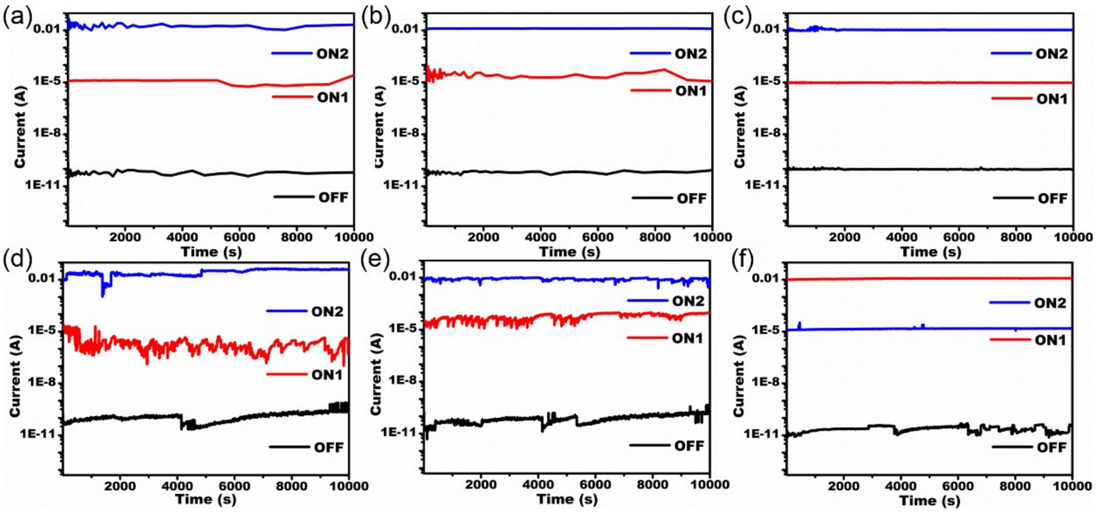

Figure S4. Characteristic retention stability of SA-Bu based memory devices on ITO (a and d), PPA-ITO (b and e) and OPA-ITO (c and f) substrates in “OFF”, “ON1” and “ON2” states under a constant voltage of –1 V at room (a, b and c) and high temperatures (d, e and f), respectively.

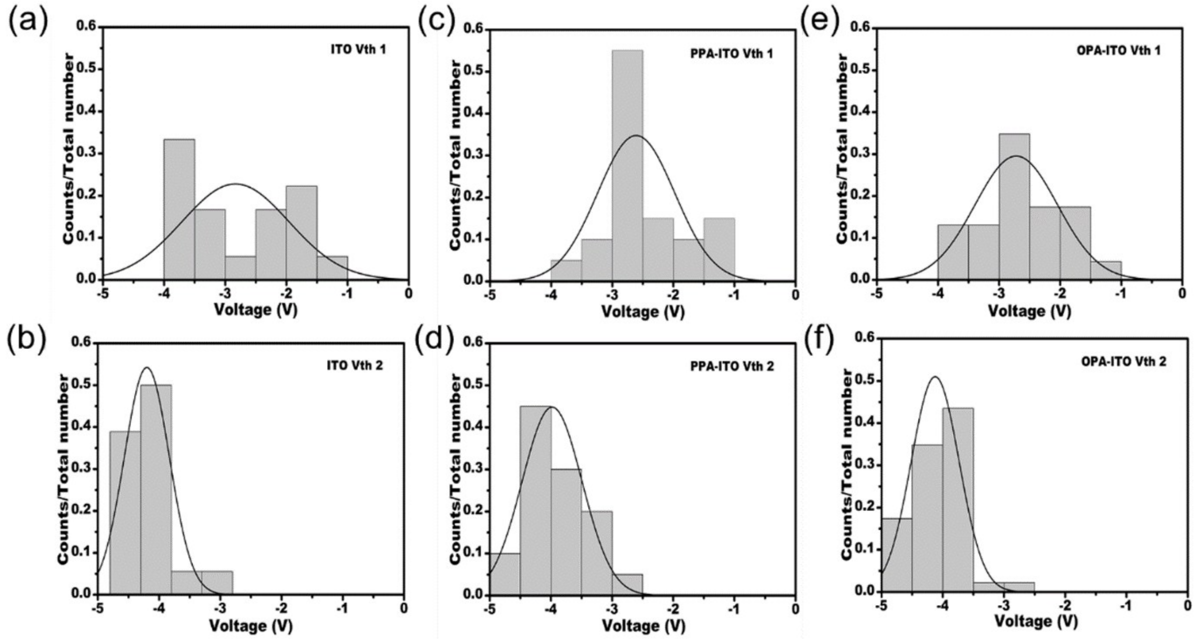

Figure S5. The distribution histograms of the Vth1 and Vth2 of ternary devices on ITO (a, b), PPA-ITO (c, d) and OPA-ITO (e, f) substrates.

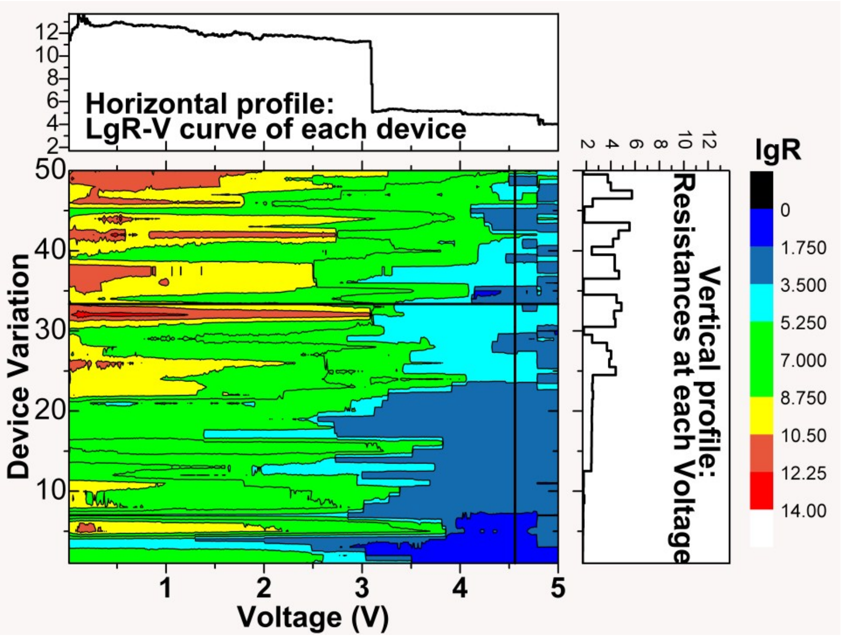

Figure S6. Two-dimensional contour map of the statistics of 50 blank-ITO-based devices. Color bar indicates the logarithm of resistances. Each horizontal line profile refers to lgR-V relationship of each individual device, while the vertical profiles refer to the lgR distribution of all devices at each particular voltage.

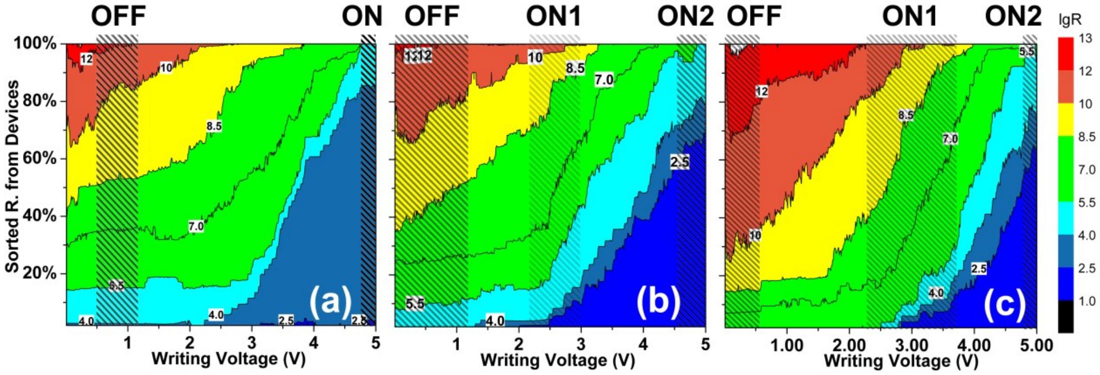

Figure S7. Two-dimensional contour map of sorted lgR distribution of three types of devices. (a) Blank ITO. (b) Phenyl-ITO based devices. (c)Octyl-based devices. The possible writing voltage windows are marked by oblique stripe shadow zone.

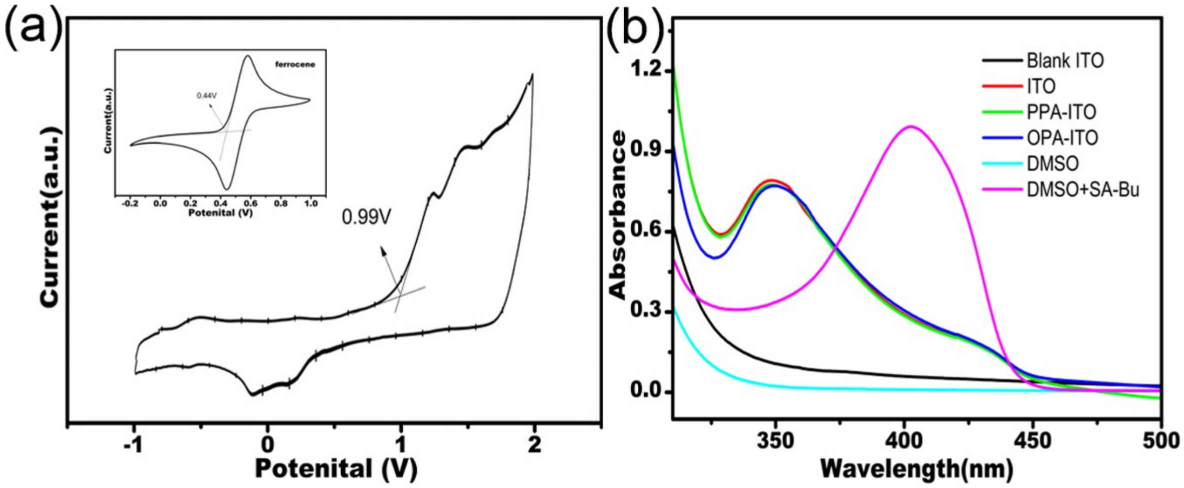

Figure S8. (a) Cyclic voltammograms of SA-Bu in DMSO solution. The scan rate is 100 mV s^{-1}. For calibration, the external ferrocene/ferrocenium (Fe/Fe^{+}) redox standard potential was measured under the same condition (0.44 eV vs. Ag/AgCl), where the absolute redox potential of Fe/Fe^{+} is assumed to be −4.80 eV to vacuum. The HOMO and LUMO were calculated according to the following equations[2, 3]: $E_{HOMO} = -[E_{ox}^{onset} + 4.8 - E_{ferrocene}]$ (eV), $E_{LUMO} = E_{HOMO} + Eg^{opt}$ (eV). Where E_{ox}^{onset} is the onsetoxidation potential vs. Ag/AgCl for the oxidation process. As shown in **Table S1**.(b)The UV-visible absorption spectra of SA-Bu solution and films evaporated on ITO, PPA-ITO, OPA-ITO substrates.

Table S1. Optical and Electrochemical Properties of SA-Bu-based Materials.

compound	λ_{max}(nm)		λ_{onset}	E_g^{opt}	E_{ox}^{onset}	HOMO	LUMO
	solution[a)]	film[b)]	(nm)	(eV)[c)]	(V)[d)]	(eV)[e)]	(eV)
SA-Bu	402.5	349.5	445.1	2.76	0.99	-5.35	-2.59

[a)] In DMSO solution at 10^{-4} M. [b)] Evaporated thin films. [c)] Estimated from the absorption onse thin films. [d)] Vs Ag/AgCl. [e)] Determined by the onset of the oxidation peak of cyclic voltammogra

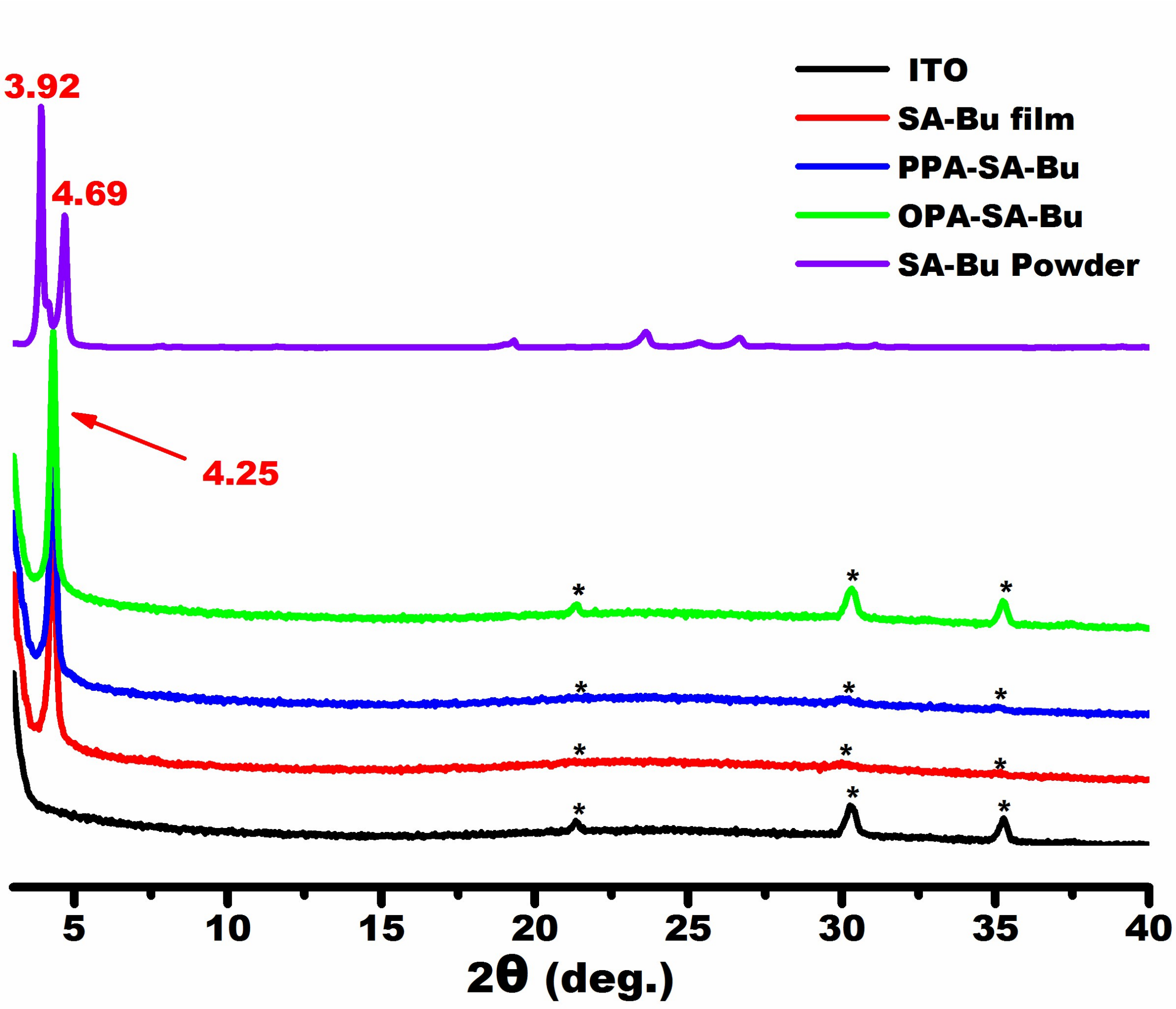

Figure S9. XRD patterns of blank ITO, SA-Bu powder, SA-Bu films on ITO substrates, PPA-ITO substrates and OPA-ITO substrates. (* The peak of ITO)

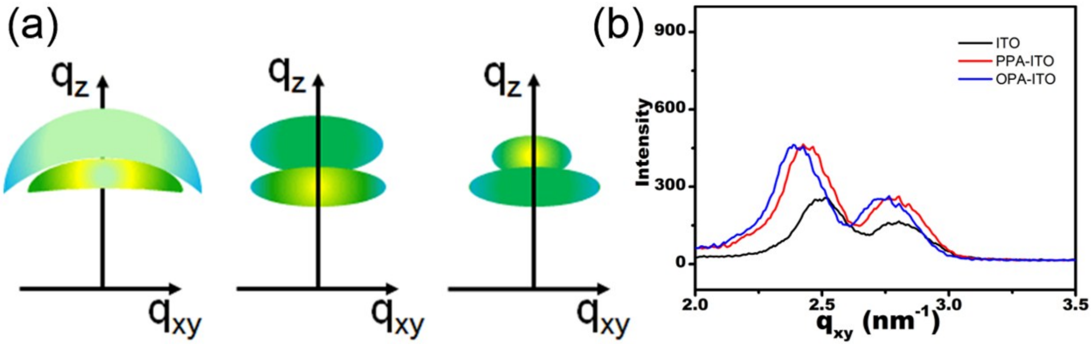

Figure S10. The films of ITO, PPA-ITO and OPA-ITO produced respectively the diffraction patterns (a) and abroad orientation distribution produced arcs of intensity (b).

Reference

[S1] A. Nagai, X. Chen, X. Feng, X. Ding, Z. Guo and D. Jiang, *Angew. Chem. Int. Ed*, 2013, **52**, 3770.

[S2] Y.-Z. Lee, X. Chen, S.-A. Chen, P.-K. Wei and W.-S. Fann, *J. Am. Chem. Soc*, 2001, **123**, 2296.

[S3] G. Liu, B. Zhang, Y. Chen, C.-X. Zhu, L. Zeng, D. S.-H. Chan, K.-G. Neoh, J. Chen and E.-T. Kang, *J. Mater. Chem*, 2011, **21**, 6027.
